# Neglected Volar Lunate Dislocation Treated With Lunate Excision and Partial Wrist Fusion

**DOI:** 10.7759/cureus.72304

**Published:** 2024-10-24

**Authors:** Vishwakarma C Hodigere, Shekhar Mishra, Prateek Behera, Manish Dwivedi, John A Santoshi

**Affiliations:** 1 Orthopaedics, All India Institute of Medical Sciences, Bhopal, Bhopal, IND

**Keywords:** neglected trauma, partial wrist fusion, scaphocapitate fusion, volar lunate dislocation, wrist injuries

## Abstract

Lunate dislocation is a rare injury caused by a fall on an outstretched hand with axial loading on the dorsiflexed wrist. These injuries are often missed and tend to present late. Median nerve compressive neuropathy is a common associated finding in such cases. We report a case of neglected lunate dislocation in a patient who presented six months after trauma with symptoms of median nerve compressive neuropathy. He was treated with lunate excision and scaphocapitate fusion. At the three-year follow-up, the patient demonstrated complete recovery of median nerve function and achieved a satisfactory functional outcome.

## Introduction

Lunate dislocations are rare but devastating injuries [1]. The usual mechanism of injury is a fall on an outstretched hand with the wrist loaded in forced dorsiflexion [2]. The lunate is often subluxated or dislocated into the carpal tunnel and may present with median nerve compression [2,3] or even rupture of flexor tendons in late-presenting cases [3,4]. Isolated lunate dislocations frequently go unrecognized at the time of the initial injury since the rest of the carpus remains aligned [5]. Almost a quarter of these cases are diagnosed late, often due to delayed presentation, improperly taken or poorly interpreted radiographs, or associated injuries that require more urgent attention [1,5-7].

When the patient presents early, the preferred treatment is reduction of the dislocated lunate, with or without ligament repair or reconstruction, and fixation [5]. However, when the patient presents beyond six to eight weeks after the injury, there is no consensus on the ideal treatment. Some surgeons advocate open reduction with ligament reconstruction, with or without augmentation, regardless of the delay [7-9], while others opt for simple excision of the lunate [2,3,10] or salvage procedures such as proximal row carpectomy (PRC) [3,7], partial wrist fusion [3,6], or total wrist fusion [3]. It is widely accepted that treating neglected, unreduced lunate dislocation is challenging and often results in a poor prognosis [3,6-8]. We present a case of neglected lunate dislocation in a patient who presented six months after trauma with symptoms of median nerve compressive neuropathy.

## Case presentation

A 44-year-old teacher presented to the orthopedics outpatient clinic with complaints of pain and restricted movement in his right wrist, along with a tingling sensation in the thumb, index, and middle fingers, and weakness in hand grip for the past six months. He reported a motor vehicle accident six months earlier, during which he sustained a head injury with loss of consciousness. After recovery, he experienced persistent pain and stiffness in his right wrist. The treating doctor reassured him that he would regain function over time. However, he continued to experience wrist pain and tingling in the thumb, index, and middle fingers, prompting him to seek further care.

**Figure 1:**
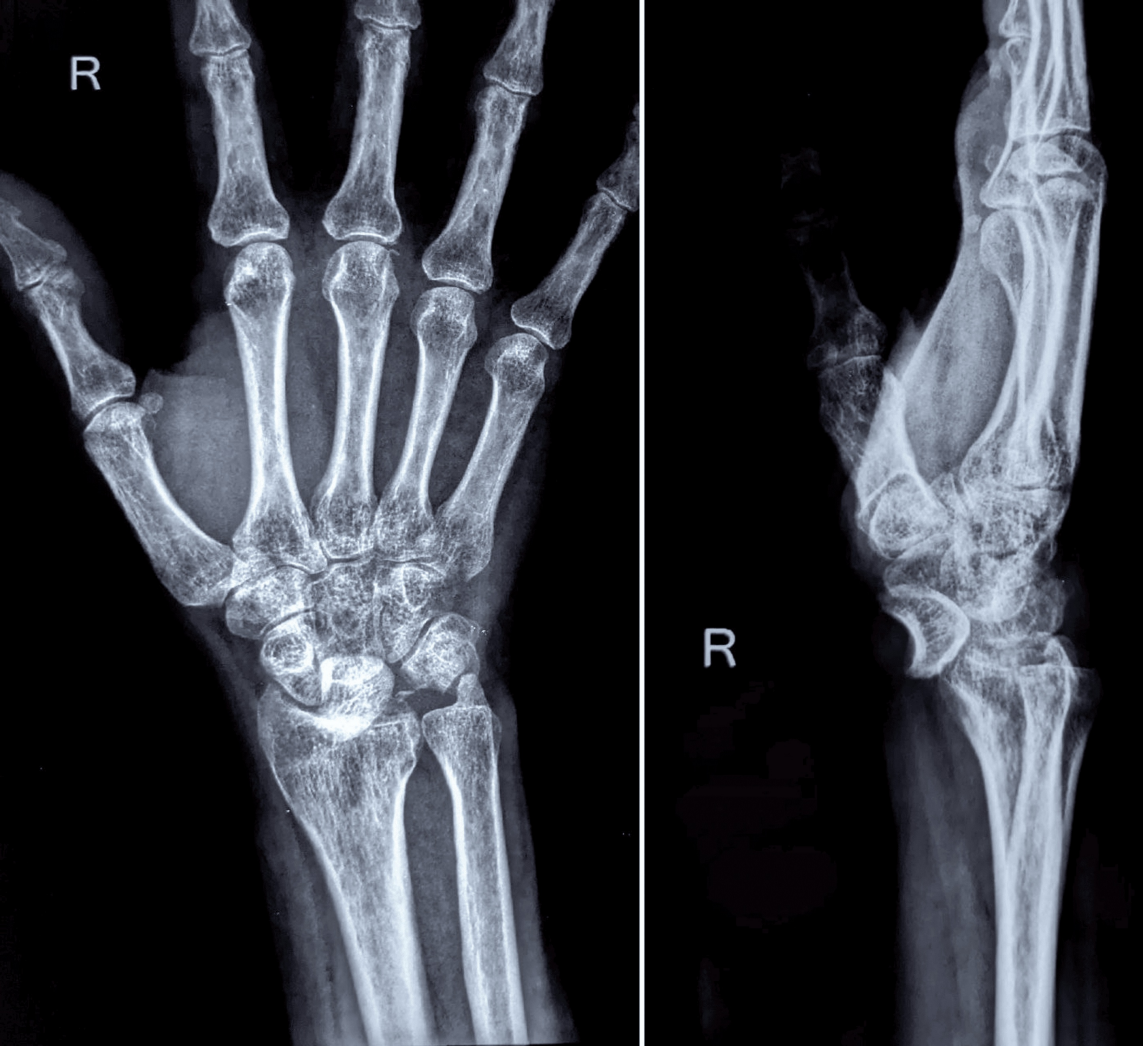
Radiographs of the right wrist showing volar dislocation of the lunate (spilled tea-cup sign) with an avulsion fracture of the triquetrum.

On examination, wasting of the thenar muscles was observed, with paresthesia over the pulp of the thumb, index, and middle fingers. The paresthesia worsened with dorsiflexion of the wrist. Active dorsiflexion of 10 degrees and palmar flexion of 20 degrees was possible in the right wrist. Radiographs revealed a volar dislocation of the lunate (spilled tea-cup sign) with an avulsion fracture of the triquetrum (Figure 1).

**Figure 2:**
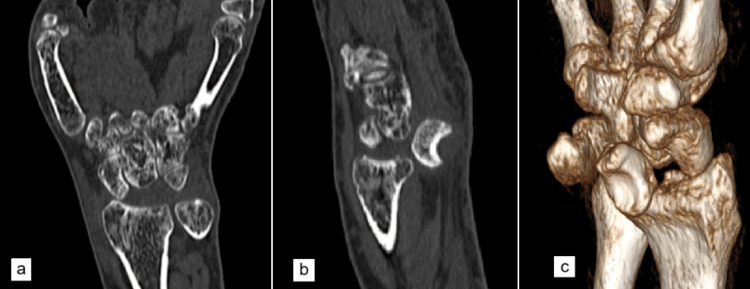
Representative CT images of the right wrist at the initial presentation showing (a) coronal section with absent lunate in the proximal carpal row, (b) sagittal section with the lunate dislocated into the carpal tunnel, and (c) three-dimensional reconstruction image of the wrist.

Computed tomography (CT) images of the wrist confirmed the radiographic findings (Figure 2).

**Figure 3:**
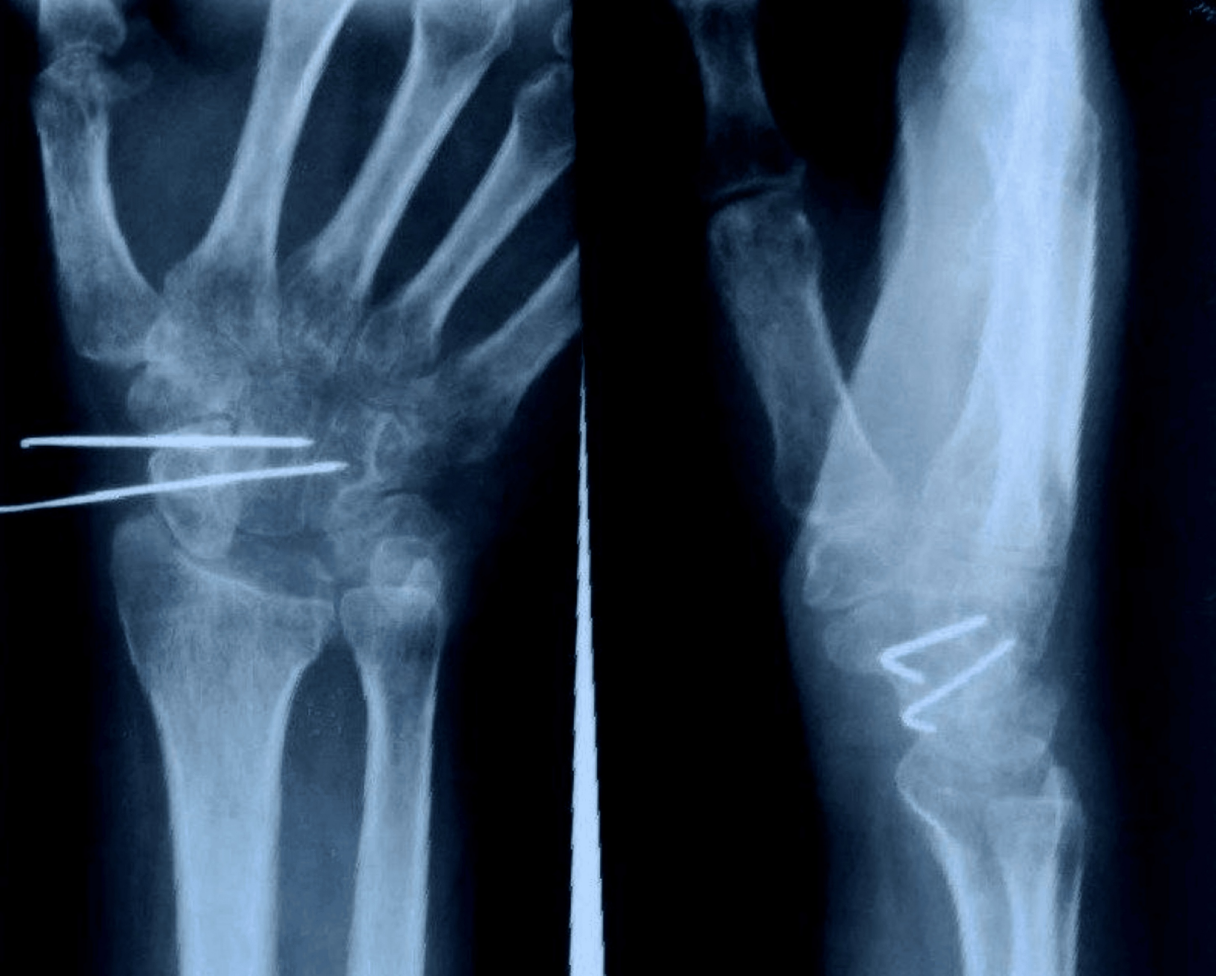
Postoperative radiographs showing Kirschner wires across the scaphocapitate joint. The lunate has been excised. The avulsion fracture of the triquetrum was left alone.

We diagnosed him with neglected volar lunate dislocation with associated median nerve compressive neuropathy of the right wrist. Given the delay in presentation and the need to prevent progressive degenerative changes in the wrist, he was offered excision of the dislocated lunate and partial carpal fusion. During surgery, through an extended anterior carpal tunnel release incision, the carpal tunnel was decompressed, and the dislocated lunate, which was found stretching the median nerve, was excised. The scaphocapitate (SC) joint was accessed through a separate dorsal approach and fixed using two Kirschner wires (Figure 3). Cancellous bone from the excised lunate was used as a graft to fill the SC fusion site.

**Figure 4:**
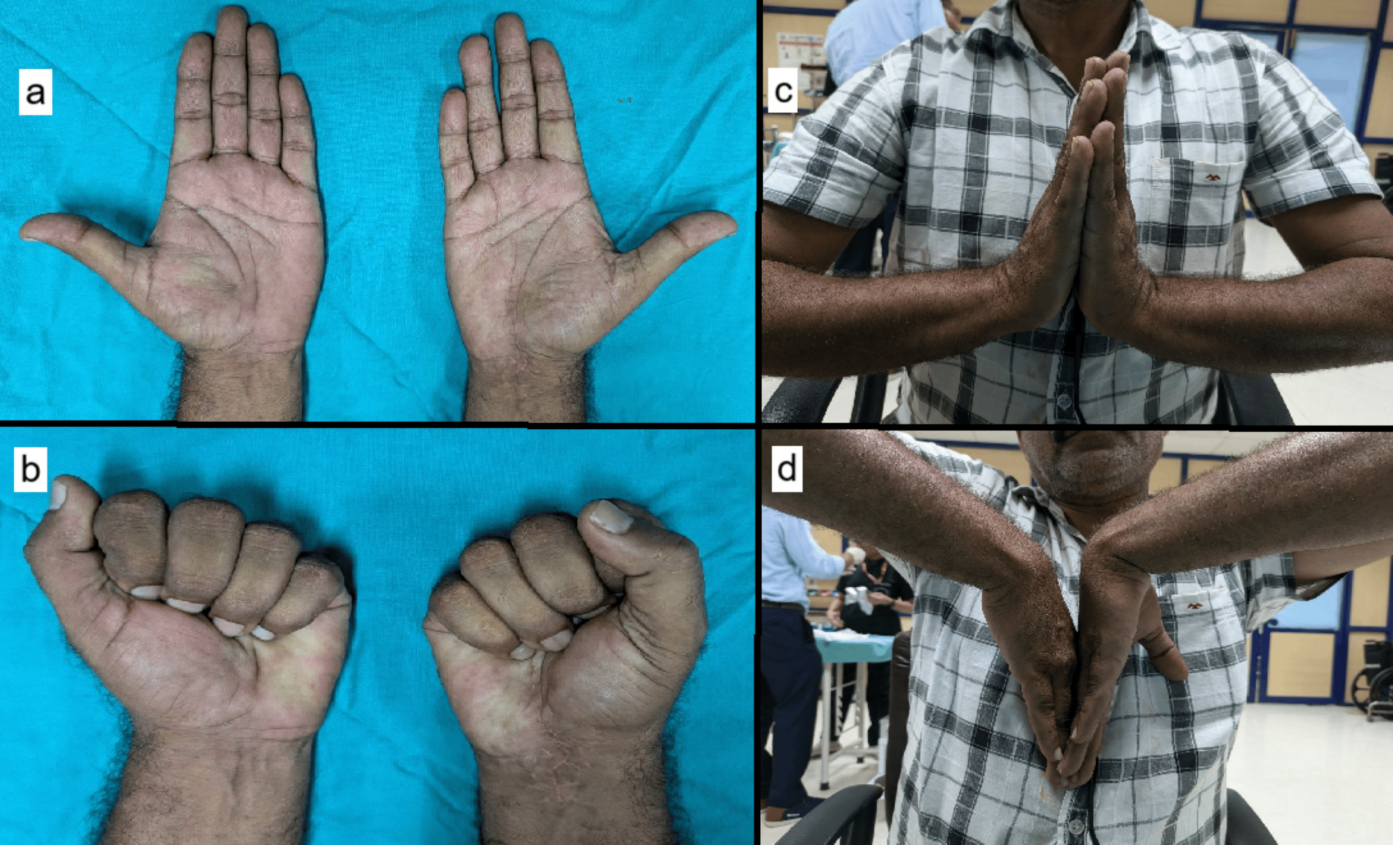
Three-year follow-up clinical images showing (a) the operative scar across the right wrist with the recovery of the bulk of the thenar muscles, (b) complete finger closure, (c) wrist dorsiflexion and (d) wrist palmar flexion.

The wrist was immobilized in a below-elbow plaster cast for six weeks. The Kirschner wires were removed after eight weeks. At the three-year follow-up, the patient had recovered median nerve function, although he reported mild paresthesia in the index finger. His range of motion included 60 degrees of dorsiflexion, 30 degrees of palmar flexion, 15 degrees of ulnar deviation, and 5 degrees of radial deviation. His grip strength was approximately 60% of that of the non-dominant hand (Figure 4).

**Figure 5:**
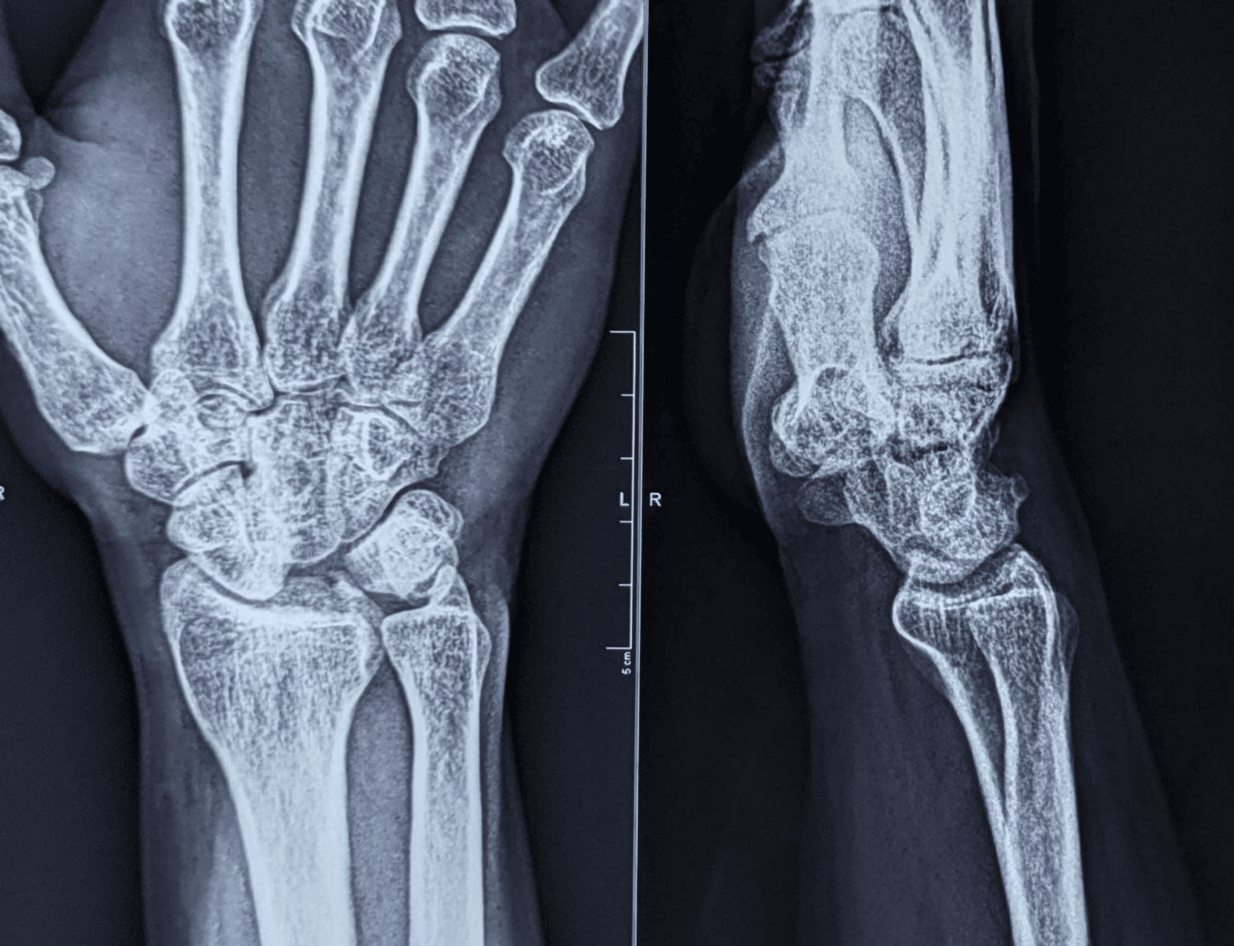
Follow-up radiographs showing solid fusion with trabeculae across the scaphocapitate articulation.

The follow-up radiographs showed solid union across the SC fusion site (Figure 5).

He had no difficulty discharging his duties as a teacher. His modified Mayo wrist score was 70, indicating a "fair" outcome. The Quick Disabilities of the Arm, Shoulder, and Hand (QuickDASH) score was 15.9, suggesting no disability [11].

## Discussion

Lunate dislocation represents stage IV of Mayfield's progressive perilunate instability and stage II of Herzberg's classification of perilunate dislocation. The lunate is palmarly subluxated or dislocated in relation to the radius [12]. The short radiolunate ligament remains the sole soft-tissue attachment to the volar aspect of the lunate, preventing its complete translocation into the carpal tunnel [13].

Since these injuries are relatively uncommon, radiological signs are often missed by emergency physicians, as they are not actively looked for, and symptoms are often attributed to wrist sprain [12,14]. Clinical assessment should be followed by carefully performed radiography, including a true lateral wrist radiograph [2].

Our patient had a trans-triquetral fracture with lunate dislocation, classified as a greater arc perilunate injury pattern [12]. The triquetral fracture appeared to be an avulsion injury of the lunotriquetral interosseous ligament (Figure 1) [3]. Li et al. [15] reported successful management of a similar injury, treated by closed reduction of the lunate dislocation with Kirschner wire fixation across the scapholunate and lunotriquetral joints.

When the patient presents early, closed reduction should be attempted, followed by surgical stabilization. However, wrist swelling may prevent a successful closed reduction [1,15]. Even with early treatment, the incidence of post-traumatic arthritis remains high [3]. Carpal instability, post-traumatic arthritis, scapholunate advanced collapse, and wrist stiffness are frequently seen in cases with delayed treatment [3,8].

Given the delayed presentation in our case, we opted for excision of the dislocated lunate and SC fusion. SC fusion is a type of partial carpal fusion used in cases of Lichtman stage IIIB Kienböck disease, scapholunate advanced collapse, scaphoid nonunion advanced collapse, or as a primary salvage procedure for complex carpal injuries where the articular surface is irreparable, or standard fixation is unlikely to be effective [16].

PRC (proximal row carpectomy) was another option that has been adopted by many authors for managing neglected lunate dislocations [3,7]. While the functional outcomes of PRC and SC fusion are comparable, SC fusion offers the advantage of maintaining carpal height and preserving radioscaphoid articulation, especially in younger patients [17]. Catalano and De Tolla [6] reported a 36-year-old patient presenting 16 years after trauma with lunate dislocation, complicated by radiolunate and lunocapitate arthritis, who was successfully treated with SC fusion. Considering our patient's age and vocation, we chose to perform lunate excision and SC fusion.

## Conclusions

Lunate dislocations are rare injuries resulting from a fall on an outstretched hand with the wrist loaded in forced dorsiflexion. These injuries are often missed at the initial presentation since the rest of the carpus remains aligned and may go undiagnosed if the clinician is unaware. A high index of suspicion is essential when such a mechanism of injury is involved, especially if the patient presents with symptoms suggestive of median nerve compressive neuropathy. While the management of acute cases is well established, treatment of delayed presentations is complex and often associated with a poor prognosis. Such cases should be managed on an individual basis. Lunate excision and partial wrist fusion should be considered viable treatment options.
